# Exploring a Life Lived with Irrational Fall Risk Appraisal

**DOI:** 10.1093/geroni/igaf122.3764

**Published:** 2025-12-31

**Authors:** Aleatha Rossler, Victoria Loerzel, Ladda Thiamwong

**Affiliations:** university of central florida, willow park, Texas, United States; University of Central Florida, Orlando, Florida, United States; University of Central Florida, Orlando, Florida, United States

## Abstract

Fall risk assessment has been focused on the physiological risk factors that can be seen, and interventions are centered on physical strengthening or assistive tools. Fear of falling (FOF) is a psychological risk factor for falls and has been classified as having worry of falling or a lack of balance confidence. Irrational Fall Risk Appraisal (FRA) is when a person has a high level of FOF with a low physical risk; however, little is known about the experience of this group in relation to falls. A qualitative descriptive design was used to learn more about their experience with FOF and any effect it may have on their daily lives. 14 older adults (OAs) with Irrational FRA were interviewed. Participants completed a semi-structured interview that was recorded and transcribed for analysis. Four themes were derived: Experiences with Falls, Acceptance of age-related decrease in ability, Perceptions of Mobility and Fears, and Awareness and Support. 13 of the participants had a prior fall. This study showed that OAs are unaware whether they are experiencing FOF or of the impact that FOF may have on them. FOF is not a common assessment done by healthcare providers and shared with the OAs, and therefore, the older population needs more education on the topic. Education for healthcare providers is needed to raise awareness of how and why to integrate these assessments into their regular practices. Combined interventions to address both psychological and physiological risks, along with community education, may provide added support to maintain independence.

